# Retinal ganglion cell autophagy: a housekeeper essential for neuronal homeostasis and survival

**DOI:** 10.1080/27694127.2026.2710457

**Published:** 2026-08-05

**Authors:** Yogapriya Sundaresan, Gulab S. Zode

**Affiliations:** a Gavin Herbert Eye Institute- Brunson Center for Translational Vision Research, Department of Ophthalmology and Visual Sciences, University of California, Irvine, CA, USA; b Department of Physiology and Biophysics, School of Medicine, University of California, Irvine, CA, USA

**Keywords:** ATG5, ATG7, autophagy, conditional knockout, neurodegeneration, neuronal homeostasis, organelle accumulation, retinal ganglion cells

## Abstract

Retinal ganglion cells (RGCs) are the sole projection neurons of the retina and the only direct link between retinal circuitry and the brain. Maintaining this lifelong connection requires constitutive autophagy to preserve organelle quality control and neuronal homeostasis. Although autophagy has been widely studied following ocular hypertension and optic nerve injury, its physiological role in healthy RGCs has remained unclear. We have recently revealed that basal autophagy is highly active in RGCs and that conditional deletion of *Atg5* or *Atg7* is sufficient to induce progressive RGC dysfunction, optic nerve degeneration, and neurodegeneration. Autophagy deficiency caused the accumulation of swollen mitochondria, distended endoplasmic reticulum, fragmented Golgi, synaptic vesicles, and incomplete autophagosomes accompanied by increased p62 and LC3B levels. These findings establish basal autophagy as an essential housekeeping mechanism that preserves organelle quality control and long-term RGC integrity.

As long-lived, post-mitotic neurons with exceptionally long axons, retinal ganglion cells (RGCs) require continuous intracellular quality control to maintain neuronal homeostasis throughout life. Sustaining visual transmission from the retina to the brain depends on efficient turnover of damaged proteins and organelles, robust mitochondrial function, and long-range axonal transport. Autophagy is uniquely positioned to coordinate these processes by removing dysfunctional intracellular components and preserving cellular integrity. Despite this fundamental role, our understanding of basal autophagy in healthy RGCs remained surprisingly limited.

Over the past decade, autophagy has been extensively investigated in experimental glaucoma and optic nerve injury. However, these studies have largely examined stress-induced autophagy following elevated intraocular pressure, optic nerve crush, or ischemic injury, leading to conflicting conclusions. Depending on the experimental model and the stage of the disease, autophagy has been reported to either protect RGCs by promoting survival or contribute to neurodegeneration by facilitating cell death. While these studies have provided important insight into injury-induced autophagic responses, they have left a more fundamental question unanswered: Is basal autophagy required to maintain healthy RGCs under physiological conditions?

Our recent study addressed this question by selectively deleting the essential autophagy genes *Atg5* or *Atg7* in mouse RGCs. We found that healthy RGCs exhibit robust basal autophagy, as suggested from prominent expression of ATG5, ATG7, LC3B, and p62 in RGCs under physiological conditions. More importantly, acute inhibition of lysosomal degradation with bafilomycin A1 rapidly increased p62 and LC3B accumulation in RGCs, providing evidence of an enhanced basal autophagic flux [1]. This finding revealed that RGCs display constitutive upregulation of autophagy, even in the absence of stress or injury, suggesting that basal autophagy is an integral component of normal RGC physiology (Figure 1).

**Figure 1.**
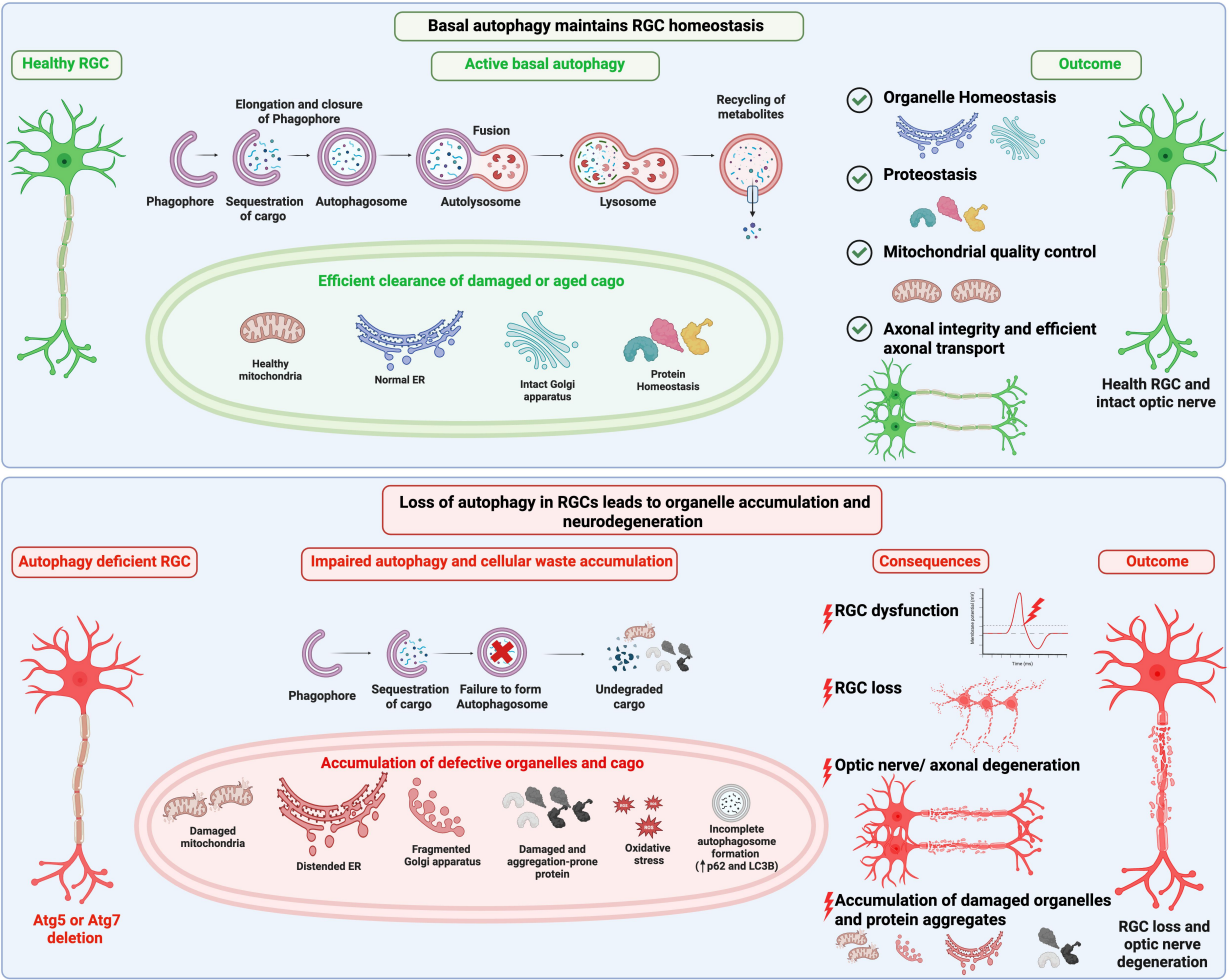
Basal autophagy is critical for RGC homeostasis and neuronal survival. Basal autophagy continuously removes and recycles dysfunctional proteins, which can form aggregates, and damaged organelles to preserve RGC homeostasis. In healthy RGCs, the efficient basal autophagic flux maintains organelle integrity, proteostasis, mitochondrial quality control, axonal transport, and neuronal function. In contrast, deletion of *Atg5* or *Atg7* blocks autophagosome formation, resulting in defective cargo degradation and accumulation of damaged mitochondria, distended endoplasmic reticulum, fragmented Golgi apparatus, protein aggregates, and immature autophagic structures, a phenotype accompanied by increased oxidative stress. These cellular defects ultimately impair RGC function, disrupt axonal integrity, promote optic nerve degeneration, and lead to progressive RGC loss.

Consistent with this notion, conditional deletion of either *Atg5* or *Atg7* was sufficient to induce progressive RGC dysfunction, optic nerve degeneration, and neurodegeneration in the absence of elevated intraocular pressure or axonal injury. One of the most striking consequences of autophagy disruption was the profound accumulation of intracellular organelles within RGC somata. Transmission electron microscopy revealed swollen mitochondria, distended endoplasmic reticulum, fragmented Golgi apparatus, synaptic vesicles, and incompletely formed autophagosomes. Immunofluorescence analysis further demonstrated increased p62 and LC3B levels, consistent with impaired autophagic clearance, together with elevated 8-OHdG levels, indicating increased oxidative stress (Figure 1). Proteomic analysis further demonstrated enrichment of proteins associated with intracellular organelles, membrane-bound compartments, cytoplasmic structures, and ribonucleoprotein complexes [1]. Together, these results indicated that basal autophagy functions as a central regulator of organelle quality control. When autophagic clearance is impaired, dysfunctional organelles and undegraded cargo progressively accumulate, ultimately overwhelming the ability of RGCs to maintain cellular homeostasis.

Mitochondria were particularly susceptible to autophagy deficiency. Degenerating RGCs and optic nerve axons accumulated swollen mitochondria, vesicular structures, and cellular debris, indicating widespread disruption of organelle homeostasis. Because RGCs possess exceptionally long axons and distal synaptic terminals, they depend on healthy mitochondria to sustain ATP production required for axonal transport, synaptic transmission, and neuronal survival. Efficient mitochondrial turnover through mitophagy is therefore probably essential for maintaining mitochondrial homeostasis. Interestingly, the optic nerve head, the earliest site where glaucomatous axonal injury occurs, is characterized by impaired axonal transport and mitochondrial accumulation in glaucoma. Although axonal transport was not directly examined in our study, we speculate that defective autophagic clearance promotes the progressive accumulation of dysfunctional mitochondria and undegraded cargo within RGC axons, reducing ATP production while increasing oxidative stress, inflammation, and cell death. This failure in mitochondrial quality control may initiate a feed-forward cycle of impaired axonal transport, mitochondrial accumulation, and progressive optic nerve degeneration, underlying a potential crucial function of basal autophagy as a critical mechanism for maintaining RGC integrity.

Our findings also provide a new perspective on previous glaucoma studies. Rather than viewing autophagy solely as a stress response that may be either protective or detrimental depending on experimental context, our work demonstrates that basal autophagy is an essential physiological process required to preserve organelle quality control, mitochondrial function, and long-term RGC survival. We propose that diminished basal autophagic capacity lowers the threshold for glaucomatous neurodegeneration by allowing dysfunctional organelles and damaged proteins to accumulate before additional pathological stressors are encountered. In this context, glaucoma may represent not only a disease caused by mechanical or metabolic stress, but also one caused by impaired intracellular quality control.

Several important questions remain. Which autophagic cargoes are most critical for maintaining RGC homeostasis, and does defective turnover of specific proteins or organelles directly initiate RGC degeneration? In addition, are the observed phenotypes caused exclusively by impaired canonical autophagy, or do they also reflect disruption of other ATG5- and ATG7-dependent pathways, including conjugation of ATG8 to single membranes (CASM)? Although our study demonstrates widespread accumulation of dysfunctional organelles, the molecular cargoes that drive neuronal dysfunction remain unknown. Defining the proteins and organelles that normally depend on constitutive autophagy for clearance, distinguishing canonical autophagy from other ATG5/ATG7-dependent processes, and determining how their accumulation disrupts mitochondrial function, axonal transport, synaptic integrity, and inflammatory signaling will provide important mechanistic insight into RGC degeneration. Addressing these questions may reveal novel therapeutic targets to preserve RGC integrity during glaucoma, aging, and other optic neuropathies.

## Data Availability

Data sharing is not applicable to this article as no data were created or analyzed.

## Abbreviations

ATG: autophagy-related
CASM: conjugation of ATG8 to single membranes
LC3B: microtubule-associated protein 1 light chain 3 beta
p62/SQSTM1: sequestosome 1
RGCs: retinal ganglion cells
8-OHdG: 8-Hydroxy-2′-deoxyguanosine
